# The catatonia syndrome: forgotten but not gone – a case report

**DOI:** 10.1192/bjo.2021.370

**Published:** 2021-06-18

**Authors:** Blerta Cenko, Spiro Milic

**Affiliations:** 1 Barnet, Enfield and Haringey Mental Health Trust; 2 Barnet Enfield and Haringey MH NHS Trust

## Abstract

**Aims:**

To highlight the presentation and treatment of catatonia in a patient with Schizophrenia.

**Background:**

Catatonia is a syndrome of altered motor behaviour accompanying many general and neurological disorders. It frequently goes unrecognized, leading to the erroneous conclusion that it is rare. Signs and symptoms of catatonia are commonly relieved by the intravenous (IV) administration of a barbiturate or benzodiazepine. If the patient does not fully respond to the sedative drug, ECT becomes the default.

**Result:**

A 61-year Caucasian male with a diagnosis of Paranoid Schizophrenia had been stable for 17 years on Clozapine. He was monitored by his GP. He resided in supported accommodation for 19 years and he was rehoused in a new borough. He was unable to obtain new prescription for Clozapine from his new GP and suffered a psychotic relapse following a period with no Clozapine and admitted under section 2 of the MHA. Clozapine was not restarted due to concerns of prolonged QTc and ectopics. Aripiprazol 15 mg and promethazine were prescribed. He was transferred to a medical ward three weeks later presenting as rigid with abnormal posturing on his bed, febrile, tachycardic and mute. He was confused, withdrawn and not responding to questions. In the medical ward he was bedbound, had high spiking temperatures, raised CK, ongoing fever.He was agitated , restless and confused with dystonic movements of arms and legs and echolalia. He developed an oral thrush, fecal impaction and was catheterised, had mittens put on due to pulling his iv cannulas. Clonazepam 2 mg QDS was prescribed, antipsychotic stopped and rehydrated. After two weeks in hospital clozapine was reintroduced and titrated accordingly. After 8 weeks Lorazepam was introduced as 1 mg QDS and he discharged to psychiatry unit on Lorazepam 1.5 mg QDS after 82 days in medical ward. He continued to be rigid and psychotic. Treatment continued with lorazepam increased up to 16 mg daily and 8 session of ECT were prescribed. Following ECT his mental state improved significantly and there was no rigidity or abnormal movements.

**Conclusion:**

Catatonia is better regarded as a movement and behavioral syndrome with particular attributes and diverse antecedents. First line of treatment is high dose of Lorazepam and second line ECT. Catatonia is a diagnosable and treatable entity.More education is needed to reinforce this message for physicians, especially in emergency departments and psychiatric facilities.

